# The Digital Lifeline: Telemedicine and Artificial Intelligence Synergy as a Catalyst for Healthcare Equity in Pakistan

**DOI:** 10.7759/cureus.54017

**Published:** 2024-02-11

**Authors:** Bilal Irfan, Aneela Yaqoob

**Affiliations:** 1 Microbiology and Immunology, University of Michigan, Ann Arbor, USA; 2 Infectious Diseases, Beaumont Hospital, Taylor, USA

**Keywords:** health-care equity, artificial intelligence in telemedicine, healthcare accessibility, telemedicine, rural healthcare delivery

## Abstract

Telemedicine emerges as a critical innovation in Pakistan, aiming to overcome the nation's unique healthcare delivery challenges, including inadequate facilities, professional scarcity, and access disparities. Examining telemedicine's potential to bridge the healthcare gap, particularly in rural and underserved regions plagued by a digital divide and infrastructural deficits, is crucial. There is a critical need for robust digital infrastructure, regulatory frameworks, and digital literacy to facilitate telemedicine adoption. By highlighting the socio-economic and logistical obstacles alongside proposed strategic interventions, the analysis suggests that telemedicine can significantly enhance healthcare accessibility, efficiency, and equity across Pakistan, offering a pragmatic solution to its pressing healthcare needs while also opening room for artificial intelligence in the landscape.

## Editorial

The push towards telemedicine in Pakistan is not merely a response to the global digital healthcare trend but a critical necessity born out of the country's unique healthcare delivery challenges. Pakistan, with its sprawling rural landscapes and densely populated urban centers, faces a myriad of healthcare delivery issues, including, but not limited to, inadequate healthcare facilities, uneven distribution of medical professionals, and financial constraints that limit access to care. Pakistan's healthcare system is further strained by a physician-to-population ratio of approximately 1:1300, significantly lower than the WHO's minimum recommended ratio of 1:1000 [1]. Telemedicine, therefore, emerges as a beacon of hope, offering a pragmatic solution to bridge these gaps, especially in underserved regions.

In rural areas and underserved regions, such as Balochistan and interior Sindh, the scarcity of healthcare infrastructure is profound. Residents often travel vast distances to access basic healthcare, a journey fraught with financial and logistical challenges. The concept of telemedicine, thus, holds immense promise in these areas, offering the potential to access medical advice and consultations without the need for physical travel. However, the realization of this potential is hampered by significant obstacles. One of the foremost challenges is the digital divide. Recent data reveals that 85.6% of users access the internet at least once a day, with 14.4% using it multiple times daily. Interestingly, internet access is predominantly from home (56.1%), followed by access through universities, work, family, or friends (15.2%), and from multiple places (12.9%) [2]. This pattern highlights the pivotal role of home-based internet connectivity in enabling telemedicine, yet it also underscores the need for broader access points to cater to those without home internet. Despite Pakistan's significant strides in improving internet connectivity, rural areas continue to grapple with inadequate internet infrastructure, making telemedical services less accessible. This technological disparity is further compounded by a lack of digital literacy among the rural population, making the adoption of telemedicine services a formidable challenge [3].

Moreover, the healthcare system in Pakistan is marred by an uneven distribution of healthcare professionals, with a significant concentration in urban centers like Karachi, Lahore, and Islamabad, leaving rural and remote areas with sparse medical expertise. The urban-to-rural doctor ratio is stark, with urban areas enjoying nearly four times the availability of healthcare professionals compared to rural settings [4]. Telemedicine could mitigate this imbalance by enabling remote consultations; however, the adoption among healthcare providers is hindered by several factors. A primary concern is the absence of a robust regulatory framework that governs telemedical practices. Without clear guidelines and standards, healthcare providers are cautious, fearing legal ramifications and questioning the efficacy of telemedicine in ensuring the quality of care.

Additionally, the socio-economic fabric of Pakistan poses unique challenges to the adoption of telemedicine. In regions where poverty prevails, and education levels are low, there is a notable lack of awareness about telemedicine. Moreover, electricity access remains unreliable in many parts of the country, with rural areas experiencing power outages for up to 18 hours a day, further complicating the use of digital technology for telemedicine [5]. The cost associated with setting up the necessary technology for telemedical consultations, such as smartphones or computers, remains out of reach for many, further limiting access to these services. This situation calls for targeted government intervention to subsidize or provide the necessary technology to underserved populations, ensuring equitable access to telemedical services.

The importance of pivoting towards telemedical care in Pakistan cannot be overstated. In a country where healthcare delivery faces numerous obstacles, telemedicine offers a viable solution to extend healthcare access, reduce costs, and improve patient outcomes. For telemedicine to achieve its full potential, however, a concerted effort is needed from all stakeholders. This includes significant investment in digital infrastructure to ensure widespread internet connectivity, the development of comprehensive telemedicine regulations to provide clarity and security for healthcare providers, and initiatives aimed at increasing digital literacy among the population. Furthermore, the integration of telemedicine into the national healthcare strategy, with a specific focus on training healthcare professionals in telemedical practices, is crucial. 

In the endeavor to enhance healthcare delivery through telemedicine in Pakistan, the integration of artificial intelligence (AI), particularly advanced conversational models like ChatGPT, presents a significant opportunity. AI-driven platforms can provide immediate, reliable medical information, acting as preliminary consultation points that guide individuals on when and how to seek further medical attention. Utilizing ChatGPT, for instance, could democratize access to health information, offering explanations on medical conditions, potential treatments, and preventive measures in a user-friendly manner. This technology can also be instrumental in overcoming the barriers posed by the digital divide, as it requires minimal internet bandwidth compared to video consultations and can be accessed through basic smartphones. By leveraging AI in telemedicine, Pakistan can make strides in not only improving access to healthcare information but also in enhancing the overall efficiency of healthcare service delivery, especially in regions where medical professionals are scarce. This approach could significantly alleviate the workload on healthcare professionals by filtering out cases that require immediate attention from those that can be managed through self-care, thus optimizing resource allocation and focusing human expertise where it's most needed.

While telemedicine presents a promising avenue for addressing healthcare delivery challenges in Pakistan, its successful implementation is contingent upon overcoming the existing technological, regulatory, and socio-economic barriers. Addressing these challenges requires a strategic, multifaceted approach that includes enhancing digital infrastructure, establishing clear regulatory frameworks, and promoting digital literacy. By prioritizing these initiatives, Pakistan can leverage telemedicine to make healthcare more accessible, efficient, and equitable across all regions, particularly in rural and underserved areas.
